# Increased Blood Pressure Variability Prior to Chronic Kidney Disease Exacerbates Renal Dysfunction in Rats

**DOI:** 10.3389/fphys.2016.00428

**Published:** 2016-09-23

**Authors:** Frederico F. C. T. Freitas, Gilberto Araujo, Marcella L. Porto, Flavia P. S. Freitas, Jones B. Graceli, Camille M. Balarini, Elisardo C. Vasquez, Silvana S. Meyrelles, Agata L. Gava

**Affiliations:** ^1^Biotechnology Graduate Program, Health Sciences Center, Federal University of Espirito SantoVitoria, Brazil; ^2^Physiological Sciences Graduate Program, Health Sciences Center, Federal University of Espirito SantoVitoria, Brazil; ^3^Morphology Department, Health Sciences Center, Federal University of Espirito SantoVitoria, Brazil; ^4^Department of Physiology and Pathology, Health Sciences Center, Federal University of ParaibaJoao Pessoa, Brazil; ^5^Pharmaceutical Sciences Graduate Program, University of Vila VelhaVila Velha, Brazil; ^6^Division of Nephrology, McMaster UniversityHamilton, ON, Canada

**Keywords:** blood pressure variability, sinoaortic denervation, chronic kidney disease, 5/6 nephrectomy, renal function

## Abstract

Increased blood pressure variability (BPV), which can be experimentally induced by sinoaortic denervation (SAD), has emerged as a new marker of the prognosis of cardiovascular and renal outcomes. Considering that increased BPV can lead to organ-damage, the goal of the present study was to evaluate the effects of SAD on renal function in an experimental model of chronic kidney disease (CKD). SAD was performed in male Wistar rats 2 weeks before 5/6 nephrectomy and the animals were evaluated 4 weeks after the induction of CKD. Our data demonstrated that BPV was increased in SAD and CKD animals and that the combination of both conditions (SAD+CKD) exacerbated BPV. The baroreflex sensitivity index was diminished in the SAD and CKD groups; this reduction was more pronounced when SAD and CKD were performed together. 5/6 nephrectomy led to hypertension, which was higher in SAD+CKD animals. Regarding renal function, the combination of SAD and CKD resulted in reduced renal plasma and blood flow, increased renal vascular resistance and augmented uraemia when compared to CKD animals. Glomerular filtration rate and BPV were negatively correlated in SAD, CKD, and SAD+CKD animals. Moreover, SAD+CKD animals presented a higher level of glomerulosclerosis when compared to all other groups. Cardiac and renal hypertrophy, as well as oxidative stress, was also further increased when SAD and CKD were combined. These results show that SAD prior to 5/6 nephrectomy exacerbates renal dysfunction, suggesting that previous augmented BPV should be considered as an important factor to the progression of renal diseases.

## Introduction

It is well-established that the maintenance of blood pressure (BP) at stable levels is a *sine qua non* condition for adequate tissue perfusion (Vasquez et al., 2012). Increased blood pressure variability (BPV) is closely associated with the development, progression and severity of cardiac, vascular, and renal organ damage (Sander et al., 2000; Sega et al., 2002; Mancia and Parati, 2003; Tatasciore et al., 2007) as well as with an augmented risk of cardiovascular and renal outcomes (Kikuya et al., 2000; Pringle et al., 2003; Hansen et al., 2010; Stolarz-Skrzypek et al., 2010). Focusing on the kidney, renal function is linearly negatively associated with BPV, and changes in this parameter, regardless the mean BP levels, may predict the development and progression of renal damage (Parati et al., 2012).

Experimentally, increased BPV can be induced by the bilateral disruption of the afferent pathway of the arterial baroreflex system (Kudo et al., 2009), also known as sinoaortic denervation (SAD). This model results in a transient elevation of BP, followed by its normalization within a few days (Osborn and England, 1990) and increased BPV (Norman et al., 1981). In rats, SAD also leads to cardiac hypertrophy with impaired diastolic and systolic function, as well as pulmonary hypertension (Flues et al., 2012). In the kidney, SAD causes significant alterations in renal structures, such as patchy focal sclerotic changes associated with glomerular and tubular atrophy, and interstitial fibrosis in the renal cortex. The interlobular and afferent arterioles adjacent to the sclerotic lesions present arteriolar remodeling characterized by VSMC proliferation and extracellular matrix deposition, leading to the luminal narrowing and occlusion (Aoki et al., 2014).

Chronic kidney disease (CKD) is a serious disorder, and its prevalence is increasing worldwide (James et al., 2010). The progressive nature of CKD and the ensuing end-stage renal disease put a substantial burden on global health-care resources (Meguid El Nahas and Bello, 2005). The classical mechanisms involved in the progression of CKD include activation of the renin-angiotensin system, increased oxidative stress, inflammatory cytokines and deposition of extracellular matrix, usually due to hypertension and/or diabetes (Pirkle and Freedman, 2013; Miranda-Díaz et al., 2016). However, increasing evidence has showed that augmented BPV can also be related to the clinical outcomes in patients with CKD (Mallamaci and Tripepi, 2013). Considering that, we hypothesized that increased BPV prior to the onset of kidney disease could accelerate the disease progression. To test this hypothesis, we evaluated the effects of SAD previously to 5/6 nephrectomy-induced CKD on renal function, glomerulosclerosis, oxidative stress, and cardiovascular parameters. Our data demonstrate that augmented BPV prior to CKD exacerbates kidney dysfunction and should be considered as an important risk factor to the progression of renal diseases.

## Materials and methods

### Animals

Experiments were conducted in male Wistar rats (8–10 weeks old), bred and maintained in the animal care facility at the Federal University of Espirito Santo, Brazil. The animals were housed in individual cages with a controlled temperature (22–23°C) and humidity (60%) and exposed to a 12:12-h light-dark cycle. All of the experimental procedures were performed in accordance with the National Institutes of Health (NIH) guidelines, and the experimental protocols were previously approved by the Institutional Animal Care and Use Committee (CEUA-UFES Protocol n°. 03/2012).

### Experimental groups

The animals were randomly divided into 4 groups: control (Sham), sinoaortic denervated (SAD), 5/6 nephrectomy (CKD) and SAD + 5/6 nephrectomy (SAD+CKD).

SAD was performed bilaterally in rats under anesthesia with a mixture of ketamine (50 mg/Kg, ip) and xylazine (10 mg/Kg, ip). After a midline neck incision, sternocleidomastoid muscles were reflected laterally, exposing the neurovascular sheath. Then, the aortic fibers traveling along the sympathetic trunk or as isolated fibers were resected as well as the superior laryngeal nerve. The superior cervical ganglia were also removed. To complete SAD, the carotid bifurcation was widely exposed, and the surrounding connective tissue was stripped off. The carotid fiber and the carotid body were sectioned (Krieger, 1964; Miao et al., 2006). The control group underwent a sham operation.

A well-established experimental model of CKD is the 5/6 nephrectomy, which was performed in this study. Two weeks after the SAD or sham procedure, the animals were anesthetized with a mixture of ketamine (50 mg/Kg, ip) and xylazine (10 mg/Kg, ip). Two of the three ramifications of the left renal artery were ligated, causing an infarction of ~2/3 of the renal mass. The right renal artery and vein were ligated and the right kidney was removed. After these procedures, the peritoneum and the skin were sutured.

### Haemodynamic measurements, baroreflex sensitivity index (BSI) and BPV determination

Four weeks after CKD induction, the animals underwent catheterization procedures. After anesthesia (ketamine 50 mg/Kg and xylazine 10 mg/Kg, ip.), a polyethylene catheter filled with heparin solution (50 UI/mL saline) was inserted into the femoral artery to measure mean arterial pressure (MAP), systolic blood pressure (SAP), diastolic blood pressure (DAP) and heart rate (HR), as well as for blood sampling. The vein was also catheterized for drug administration. The rats were allowed to recover during a 24-h period after the catheterization. To record arterial pressure, the arterial catheter was connected to a pressure transducer (Cobe Laboratories, USA) plugged into a pressure-processor amplifier and data acquisition system (MP100, Biopac Systems, USA). A 45-min recording of MAP, SAP, DAP, and HR was obtained from conscious and freely moving rats. The BPV was quantified using the standard deviation of MAP during the recorded period.

The effectiveness of SAD was confirmed by testing the reflex heart rate responses to changes in arterial pressure during intravenous injections of phenylephrine (0.25–32.0 μg/kg) and sodium nitroprusside (0.05–1.6 μg/kg), in order to achieve changes in blood pressure ranging from 5 to 50 mmHg. For each animal, BSI was calculated using the mean of changes in HR/mean of changes in MAP elicited by the different doses of phenylephrine and sodium nitroprusside. BSI for each group was calculated by averaging the BSI of the all evaluated animals.

### Renal function studies

Renal function was determined using inulin (IN) and sodium para-aminohippurate (PAH) clearance to estimate the glomerular filtration rate (GFR) and renal plasma flow (RPF), respectively (Smith et al., 1945). Four weeks after CKD induction, a separate set of animals were anesthetized with sodium thiopental (50 mg/Kg ip.), the trachea was catheterized with a polyethylene tube (PE-90) to facilitate breathing, and a catheter (PE-240) was introduced into the bladder for urine sampling. The arterial catheter was connected to a pressure transducer (Cobe Laboratories, USA) plugged into a pressure-processor amplifier and data acquisition system (MP100, Biopac Systems, USA) for continuous monitoring of MAP, SAP, DAP, and HR. The venous catheter was connected to an infusion pump (0.1 mL/min) and a saline solution (0.9%) containing 3% of mannitol was infused over 30 min. After this stabilization period, the animals received an intravenous injection of prime solution containing IN (300 mg/Kg) and PAH (6.66 mg/Kg) and were maintained on a continuous infusion of saline (0.9%) containing IN (15 mg/mL), PAH (4 mg/mL) and mannitol (3%) until the end of the experiment. At 30-min intervals, urine and blood samples were taken, for a total of 4 samples. Haematocrit was measured using a heparinized capillary tube. Plasma and urinary IN and PAH concentrations were measured using a colorimetric assay (Rocco et al., 2008). Blood samples were also used for plasma urea quantification through spectrophotometry.

Renal blood flow (RBF) and renal vascular resistance (RVR) were determined as previously described (Magalhães et al., 2006). Briefly, RBF was calculated by the equation RBF = RPF/(1- haematocrit), and RVR was calculated using the equation RVR = MAP/RBF.

In order to be able to correlate BPV with renal function, some animals were submitted to both procedures. For these animals, BPV was determined as above mentioned and inulin and PAH clearance was performed in the next day. Due to the high mortality rate when the two procedures were performed, especially in the SAD+CKD group, the number of animals in correlation analysis is limited to 4–6.

Urinary water excretion (24 h) was obtained using a metabolic cage to avoid the effects of anesthesia and/or mannitol required to perform renal haemodynamic evaluation. By the end of the treatment, a separated group of animals were placed on metabolic cages during a 24-h accommodation period, followed by urine sampling also during 24 h.

### Quantification of superoxide production

Reactive oxygen species generation was performed in blood cells using flow cytometry. Briefly, blood samples were lysed with lysing buffer 1X (Becton Dickinson) for 10 min at 37°C to remove erythrocytes. The cell suspension was then washed twice in phosphate-buffered saline (PBS) plus 1% Foetal Bovine Serum (FBS) for 10 min and centrifuged at 1200 rpm; and the supernatant was discarded. The cells were collected and resuspended in 1 mL PBS for flow cytometry analysis. For intracellular superoxide anion generation measurements, DHE (160 mM) was added to the cell suspension (10^6^ cells), which was then incubated at 37°C for 30 min in the dark. Samples were treated with 10 mM doxorubicin for 5 min to create oxidative stress without cell toxicity for the positive control, and the negative control cells were incubated with ethanol. After washing and resuspending in PBS, the cells were maintained on ice for immediate detection by flow cytometry (FACSCanto II, Becton Dickinson, San Juan, CA, USA). Data were analyzed using FACSDiva software (Becton Dickinson). For DHE fluorescence quantification, samples were acquired in duplicate, and 10,000 events were used for each measurement. Red fluorescence was detected between 564 and 606 nm using a 585/42 bandpass filter. Data are expressed as the median fluorescence intensity.

### Cardiac and kidney hypertrophy and glomerular collagen deposition

At the end of the experiments, the animals were euthanized with an overdose of sodium thiopental and perfused via the left ventricle with Krebs-Hepes buffer (pH 7.4). The left kidney and the heart were removed, cleaned of connective tissue and weighed. To generate the hypertrophy index, the ratio of kidney or heart weight to body weight was calculated. Immediately after being weighed, the kidneys were cut longitudinally, fixed in Bouin solution and then embedded in paraffin. Five micrometer-thick sections were obtained and stained with Masson' trichrome for glomerular collagen deposition quantification. The glomeruli were photographed for later analysis. Images were captured with color video camera (VKC150; Hitachi, Tokyo, Japan) connected to a microscope (AX70; Olympus, Center Valley, PA) and analyzed with a specific image program (2100 Leica EWS; Leica, Wetzlar, Germany) by a person blinded to the experimental groups. To determine glomerular sclerosis, at least 30 glomeruli were analyzed in Masson's trichrome-stained sections using the Image J program. The mean of the glomerular-stained areas (%) was used to determine the collagen deposition for each animal. The glomerulosclerosis index was determined using a semi-quantitative scale based on % stained area of the glomerulus: (1) 0–25; (2) 25–50; (3) 50–75; and (4) >75%.

### Statistical analysis

Values are expressed as means ± S.E.Ms. Statistical comparisons between the different groups were performed by Student's *t*-test or two-way analysis of variance (ANOVA) followed by Bonferroni's *post-hoc* test. The correlation between BPV and GFR variables was examined using Pearson's correlation coefficient. Statistical significance was assessed using a linear regression model. The statistical analyses were performed using Prism software (Prism 5, GraphPad Software, Inc., San Diego, CA, USA). A value of *p* < 0.05 was regarded as statistically significant.

## Results

### Haemodynamic measurements, baroreflex sensitivity index (BSI) and BPV determination

Table 1 summarizes the results of systolic (SAP), diastolic (DAP), and mean (MAP) arterial pressure and heart rate (HR) in conscious animals 24 h after the catheterization surgery. No differences were observed in any parameters between sham and SAD group. On the other hand, CKD animals presented a greater SAP, DAP, and MAP when compared to the sham and SAD. Interestingly, these changes were exacerbated in the SAD+CKD animals, which also displayed an increased HR.

**Table 1:** **Haemodynamic measurements**.

**Parameters**	**Groups**			
	**Sham (7)**	**SAD (7)**	**CKD (7)**	**SAD+CKD (6)**
MAP (mmHg)	107 ± 1	109 ± 1	154 ± 3^a^^b^	182 ± 7^a^^b^^c^
DAP (mmHg)	80 ± 2	83 ± 3	128 ± 10^a^^b^	145 ± 10^a^^b^^c^
SAP (mmHg)	140 ± 2	135 ± 3	184 ± 10^a^^b^	224 ± 10^a^^b^^c^
HR (bpm)	344 ± 5	366 ± 6	356 ± 8	387 ± 16^a^

*SAP, systolic arterial pressure; DAP, diastolic arterial pressure; MAP, mean arterial pressure; HR, heart rate. All values are expressed as means ± SEMs. The number in parentheses represents the number of animals in each group*.

a *p < 0.05 vs. Sham*;

b *p < 0.05 vs. SAD*;

c *p < 0.05 vs. CKD*.

**Figure 1:**
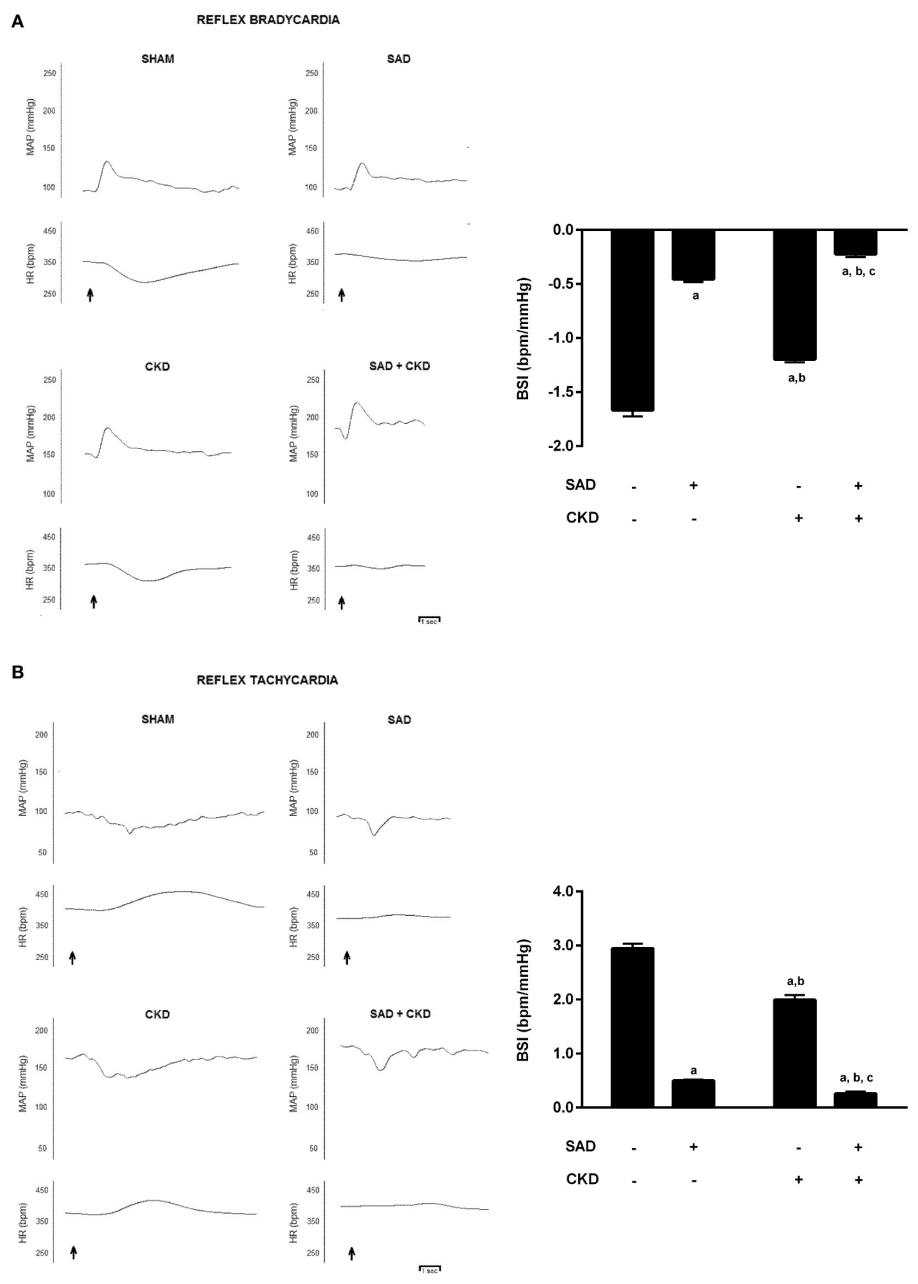
**Typical recordings of phenylephrine-induced bradycardic responses (A) and sodium nitroprusside-induced tachycardic responses (B) and quantification of baroreflex sensitivity index (BSI) (A,B) in all studied groups**. The graphs show that both SAD and CKD decrease the BSI. However, this condition is aggravated when these two situations are concomitant. Values are means ± SEMs. All groups have *n* = 5. ^a^*p* < 0.05 vs. Sham group; ^b^*p* < 0.05 vs. SAD group; ^c^*p* < 0.05 vs. CKD group. Two-way ANOVA.

The results of the baroreflex sensitivity index (BSI) determination are displayed in Figure 1. Figure 1A shows typical recordings of phenylephrine-induced bradycardic responses (left) and BSI quantification (right) in all studied groups. As expected, the BSI was significantly reduced in the SAD group (−0.45 ± 0.03 bpm/mmHg, *p* < 0.01) when compared to the sham animals (−1.7 ± 0.06 bpm/mmHg). CKD animals also displayed a slightly reduction in BSI (−1.20 ± 0.03 bpm/mmHg, *p* < 0.01). The combination of SAD and CKD led to a greater reduction in BSI (−0.22 ± 0.03 bpm/mmHg, *p* < 0.01) when compared to all other groups. Figure 1B shows typical recordings of sodium nitroprusside-induced tachycardic responses (left) and BSI quantification (right) in all studied groups. Like in the bradycardic responses, SAD (0.50 ± 0.02 bpm/mmHg, *p* < 0.01) and CKD (2.00 ± 0.09 bpm/mmHg, *p* < 0.01) animals presented a diminished BSI when compared to the sham group (2.93 ± 0.08 bpm/mmHg). Once again, the combination of SAD and CKD worsened this parameter (0.26 ± 0.03 bpm/mmHg, *p* < 0.01).

**Figure 2:**
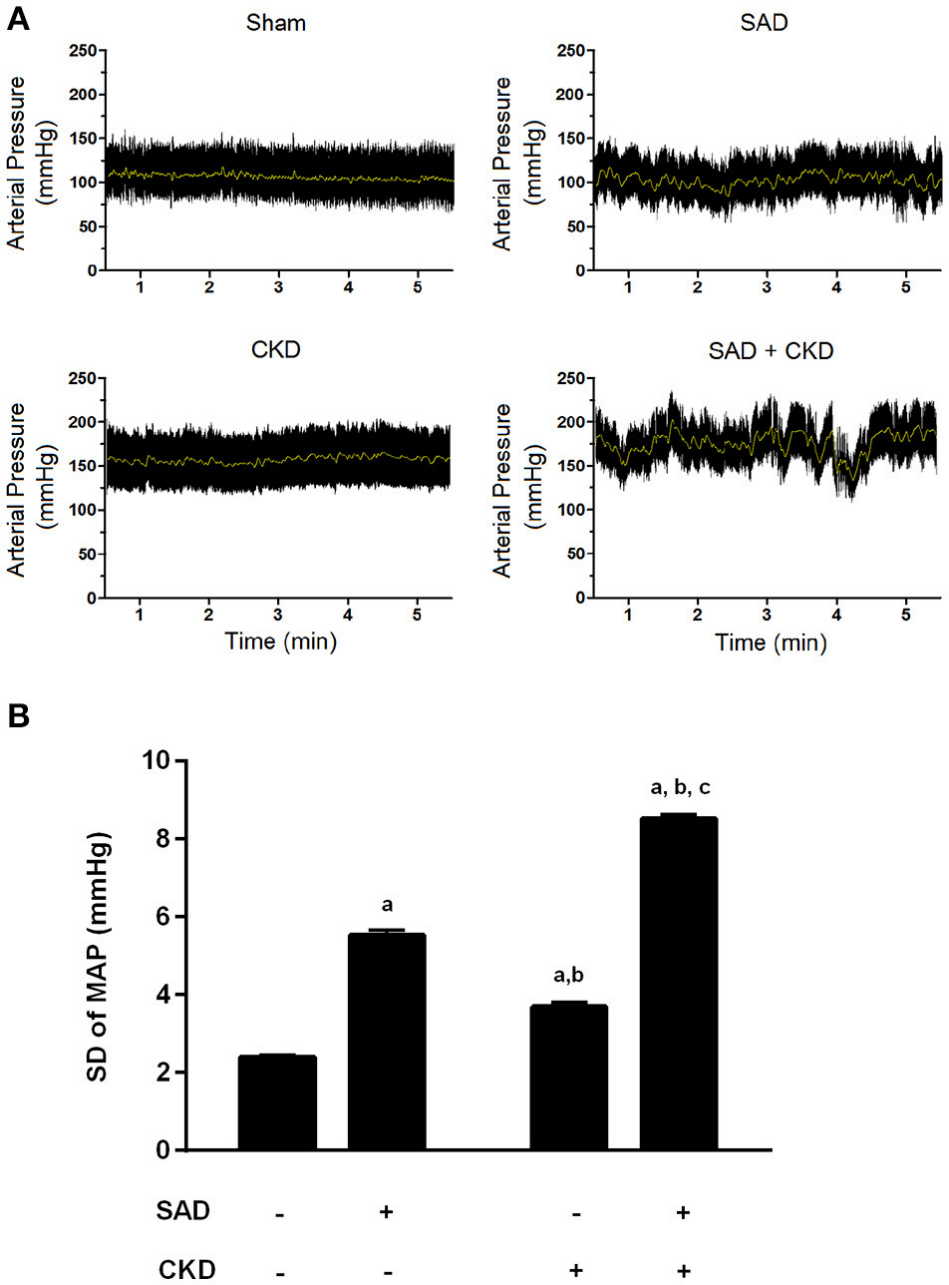
**Blood pressure variability (BPV) determined through the standard deviation of mean arterial pressure (MAP). (A)** Typical recordings showing the arterial pressure measurements at resting conditions. The black tracing represents pulsatile pressure and the gray line represents MAP. Note that BPV is remarkably enhanced when SAD and CKD are concomitant. **(B)** Bar graph showing the quantification of the SD of MAP in all studied groups. Once again, both SAD and CKD resulted in augmented BPV, and the association of these conditions caused an even more pronounced increase in BPV. Sham and SAD *n* = 4, CKD and SAD+CKD *n* = 5. Values are means ± SEMs. ^a^*p* < 0.05 vs. Sham group; ^b^*p* < 0.05 vs. SAD group; ^c^*p* < 0.05 vs. CKD group. Two-way ANOVA.

We also evaluated BP variability using the standard deviation (SD) of MAP. As demonstrated in Figure 2, the SAD animals presented an increased SD of MAP (5.53 ± 0.13 mmHg, *p* < 0.01) when compared to the sham group (2.40 ± 0.05 mmHg). CKD also augmented the SD of MAP (3.69 ± 0.12 mmHg, *p* < 0.01); however, when CKD was combined with SAD, the increase in BP variability was more pronounced (8.51 ± 0.12 mmHg, *p* < 0.01). In Figure 2, we can also observe the typical recordings of MAP during resting state in all studied groups. Note that BP variability is significantly increased in the SAD+CKD animals when compared to all other groups.

### Renal function evaluation

**Figure 3:**
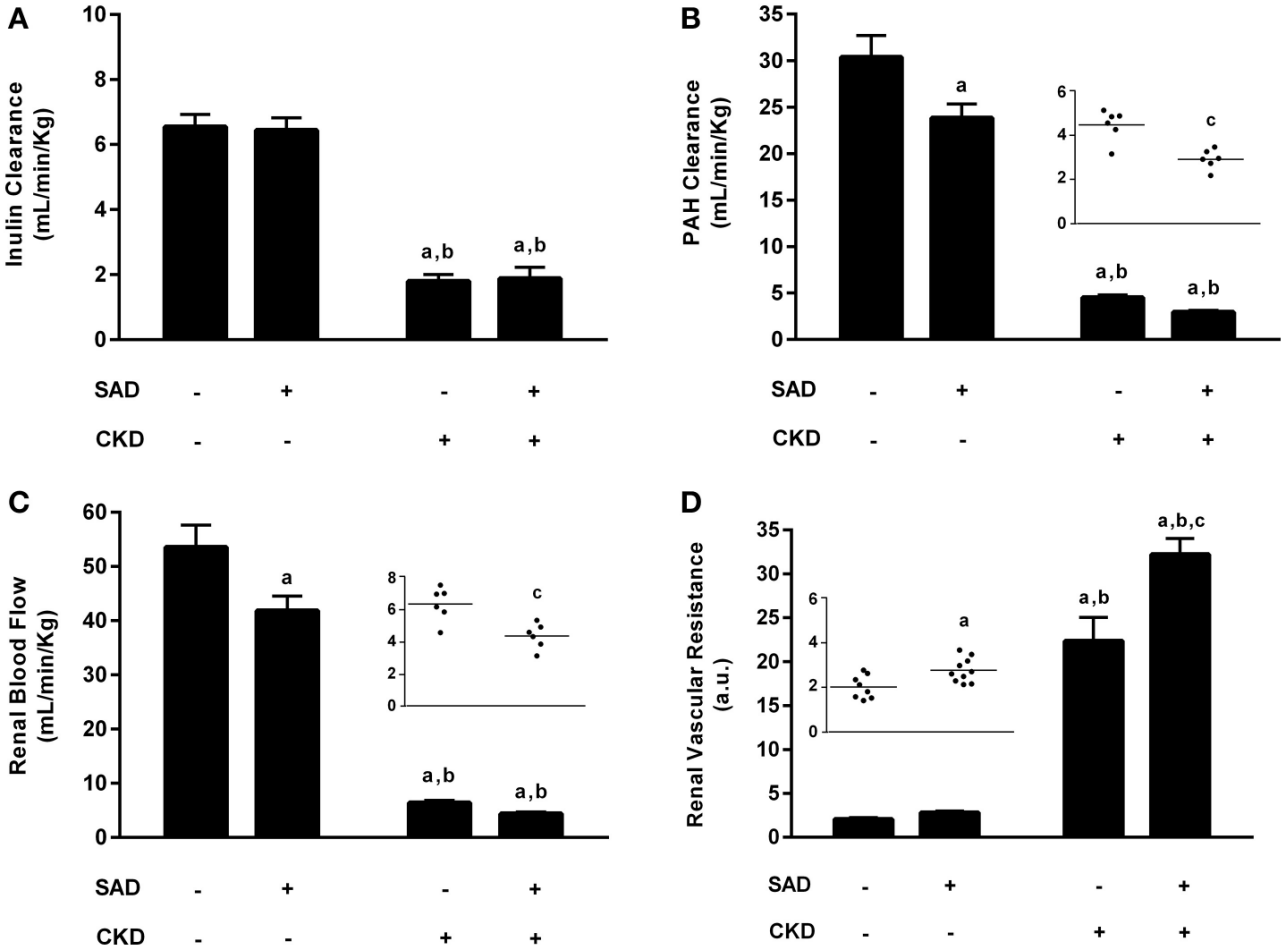
**Renal function evaluation. (A)** Glomerular filtration rate (GFR) determined through inulin clearance. As expected, CKD resulted in a marked reduction in GFR, which was not aggravated by its association with SAD. **(B)** Renal plasma flow (RPF) determined using PAH clearance. Both the SAD and CKD groups presented a reduced RPF, which was more pronounced in CKD animals. The association of SAD and CKD exacerbated this condition. **(C)** Renal blood flow (RBF) in all studied groups. Once again, SAD and CKD animals also presented reduced RBF; this reduction was greater in animals that underwent 5/6 nephrectomy. The association of SAD and CKD worsened this parameter. **(D)** Renal vascular resistance (RVR) in all studied groups. In accordance with the previous results, RVR was augmented in SAD and CKD animals; this increase was more pronounced in the CKD group. When SAD and CKD coexist, the rise in RVR is exacerbated. Sham *n* = 8, SAD *n* = 10, CKD and SAD+CKD *n* = 5. Values are means ± SEMs. ^a^*p* < 0.01 vs. Sham group; ^b^*p* < 0.01 vs. SAD group; ^c^*p* < 0.01 vs. CKD group. Two-way ANOVA. Two-way ANOVA, Student's *t*-test.

We also evaluated the effects of BPV on renal function parameters, including GFR, RPF, RBF and RVR (Figure 3). Inulin clearance results (Figure 3A) demonstrated that GFR was not modified by SAD (Sham: 6.5 ± 0.4 vs. SAD: 6.6 ± 0.4 mL/min/Kg). As expected, CKD rats presented a pronounced reduction in GFR (1.8 ± 0.2 mL/min/Kg, *p* < 0.01), which was not worsened by the combination with SAD (1.9 ± 0.4 mL/min/Kg, *p* < 0.01). RPF (Figure 3B), determined using PAH clearance, was decreased in SAD rats (23.7 ± 1.4 mL/min/Kg, *p* < 0.05) when compared to sham animals (27.9 ± 2.3 mL/min/Kg). Once again, PAH clearance was reduced in CKD rats (4.5 ± 0.3 mL/min/Kg, *p* < 0.01); however, SAD+CKD group presented a further reduction in RPF (2.9 ± 0.2 mL/min/Kg, *p* < 0.01). To calculate the RBF, we also quantified the haematocrit. SAD rats presented normal haematocrit (39 ± 2%) when compared to sham group (43 ± 2%). CKD animals demonstrated a decrease in this parameter (29 ± 1%, *p* < 0.01), which was not worsened by SAD (32 ± 1%, *p* < 0.01). The RBF analysis (Figure 3C) followed the same pattern as the RPF results. SAD resulted in reduced RBF (Sham: 53.3 ± 4.0 vs. SAD: 41.4 ± 2.5 mL/min/Kg, *p* < 0.05). CKD group displayed a marked decline in RBF (6.4 ± 0.4 mL/min/Kg, *p* < 0.01), which was worsened by combination with SAD (3.5 ± 0.7 mL/min/Kg, *p* < 0.01). As expected, based on the renal blood flow results, RVR (Figure 3D) was slightly increased in SAD rats (2.8 ± 0.2 a.u., *p* < 0.05) compared to sham animals (2.1 ± 0.1 a.u.). CKD group presented an enhanced RVR (22.4 ± 2.7 a.u., *p* < 0.01), with a further increase in SAD+CKD animals (32.2 ± 1.8 a.u., *p* < 0.01). Urinary water excretion was augmented in SAD (25.2 ± 0.7 mL/24 h, *p* < 0.05) group when compared to sham (12.5 ± 1.3 mL/24 h) animals. Experimental induction of CKD led to a further increase on urine excretion (38.3 ± 3.2 mL/24 h, *p* < 0.01), which was much more prominent in SAD+CKD (57.4 ± 6.0 mL/24 h, *p* < 0.01) group.

**Figure 4:**
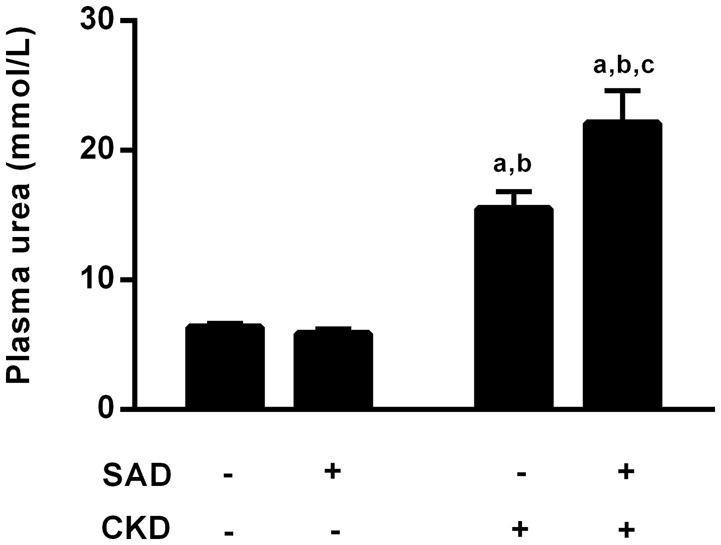
**Plasma values of urea (mmol/L)**. Sinoaortic denervation did not modify uraemia. As expected, CKD animals presented hyperuraemia, which was more pronounced in the SAD+CKD group. Sham and SAD *n* = 4, CKD *n* = 7, SAD+CKD *n* = 8. Values are means ± SEMs. ^a^*p* < 0.05 vs. Sham group; ^b^*p* < 0.05 vs. SAD group; ^c^*p* < 0.05 vs. CKD group. Two-way ANOVA.

Corroborating the inulin and PAH clearance results, the plasma urea levels are demonstrated in Figure 4. Sinoaortic denervation did not change uraemia (5.9 ± 0.4 mmol/L) compared to the sham group (6.4 ± 0.3 mmol/L). As expected, CKD animals presented hyperuraemia (15.5 ± 1.3 mmol/L, *p* < 0.01), which was aggravated in the SAD+CKD group (22.1 ± 2.5 mmol/L, *p* < 0.01).

**Figure 5:**
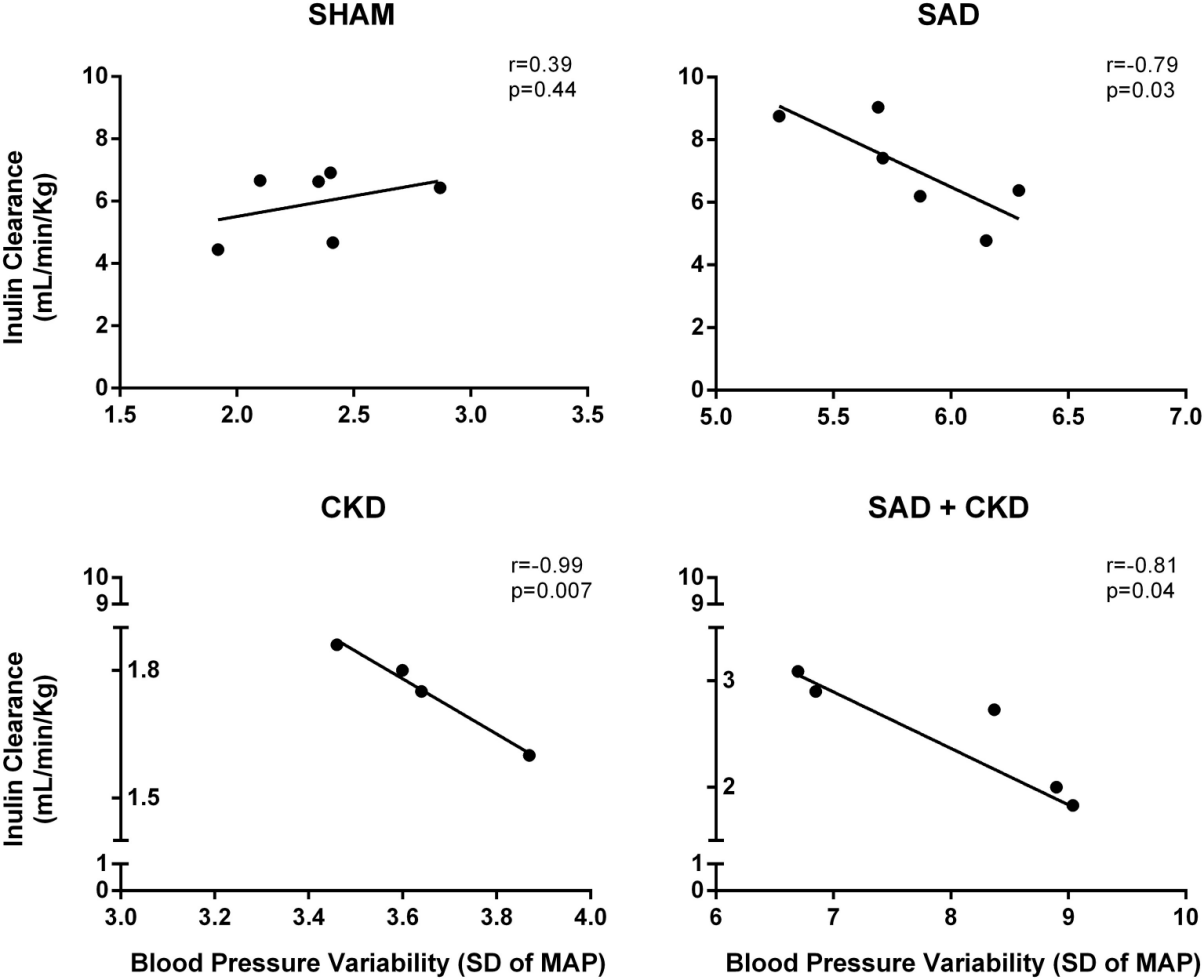
**Scatterplot linear regression and correlation analysis between glomerular filtration rate (GFR) and blood pressure variability (BPV)**. Data show a negative relationship of GFR and BPV in SAD, CKD and SAD+CKD group, but not in sham group. Sham and SAD *n* = 6, CKD *n* = 4, and SAD+CKD *n* = 5. The correlation between variables was determined using Pearson's correlation coefficient and a linear regression model.

Although SAD did not modify inulin clearance, the correlation analysis between BPV and GFR demonstrated a negative relationship between these parameters in the SAD (*r* = −0.79, *p* < 0.05), CKD (*r* = −0.99, *p* < 0.05) and SAD+CKD (*r* = −0.81, *p* < 0.05) groups (Figure 5).

### Quantification of superoxide production

**Figure 6:**
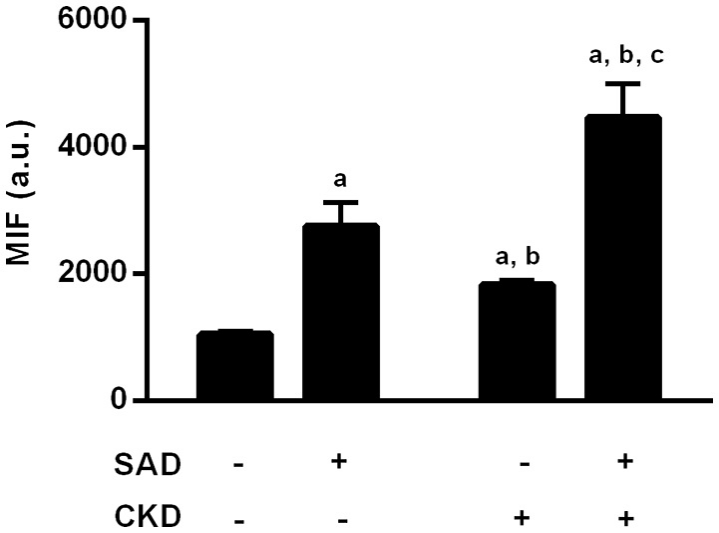
**Quantification of reactive oxygen species (ROS) formation through DHE fluorescence**. Both SAD and CKD resulted in increased ROS generation, and this condition was aggravated when SAD and CKD were associated Sham and SAD *n* = 6, CKD and SAD+CKD *n* = 5. Values are means ± SEM. ^a^*p* < 0.05 vs. Sham group; ^b^*p* < 0.05 vs. SAD group; ^c^*p* < 0.05 vs. CKD group. Two-way ANOVA.

Superoxide production was assessed using flow cytometry with DHE (median of fluorescence intensity). As illustrated in Figure 6, SAD caused an increase in ROS generation (2757 ± 374, *p* < 0.01) when compared to the sham group (1045 ± 52). CKD also resulted in augmented superoxide production (1830 ± 66, *p* < 0.01), which was worsened in the SAD+CKD animals (4468 ± 529, *p* < 0.01).

### Cardiac and kidney hypertrophy and glomerular collagen deposition

Table 2 presents the results of cardiac and kidney hypertrophy. SAD and CKD groups showed an increased heart weight/body weight ratio when compared to sham group. The cardiac hypertrophy was worsened in SAD+CKD group. The kidney weight/body weight ratio was elevated only in SAD+CKD animals.

**Table 2:** **Body weight, cardiac, and kidney hypertrophy**.

**Parameters**	**Groups**			
	**Sham (6)**	**SAD (5)**	**CKD (9)**	**SAD+CKD (6)**
Body Weight (g)	420 ± 8	373 ± 18^a^	358 ± 10^a^	296 ± 18^a^^b^^c^
CW/BW (mg/g)	3.11 ± 0.14	3.81 ± 0.10^a^	3.70 ± 0.14^a^	5.29 ± 0.19^a^^b^^c^
KW/BW (mg/g)	3.91 ± 0.10	4.32 ± 0.24	4.44 ± 0.22^a^	6.80 ± 0.47^a^^b^^c^

*CW/BW, cardiac weight to body weight ratio; KW/BW, kidney weight to body weight ratio. All values are expressed as means ± SEMs. The number in parentheses represents the number of animals in each group*.

a *p < 0.05 vs. Sham*;

b *p < 0.05 vs. SAD*;

c *p < 0.05 vs. CKD*.

**Figure 7:**
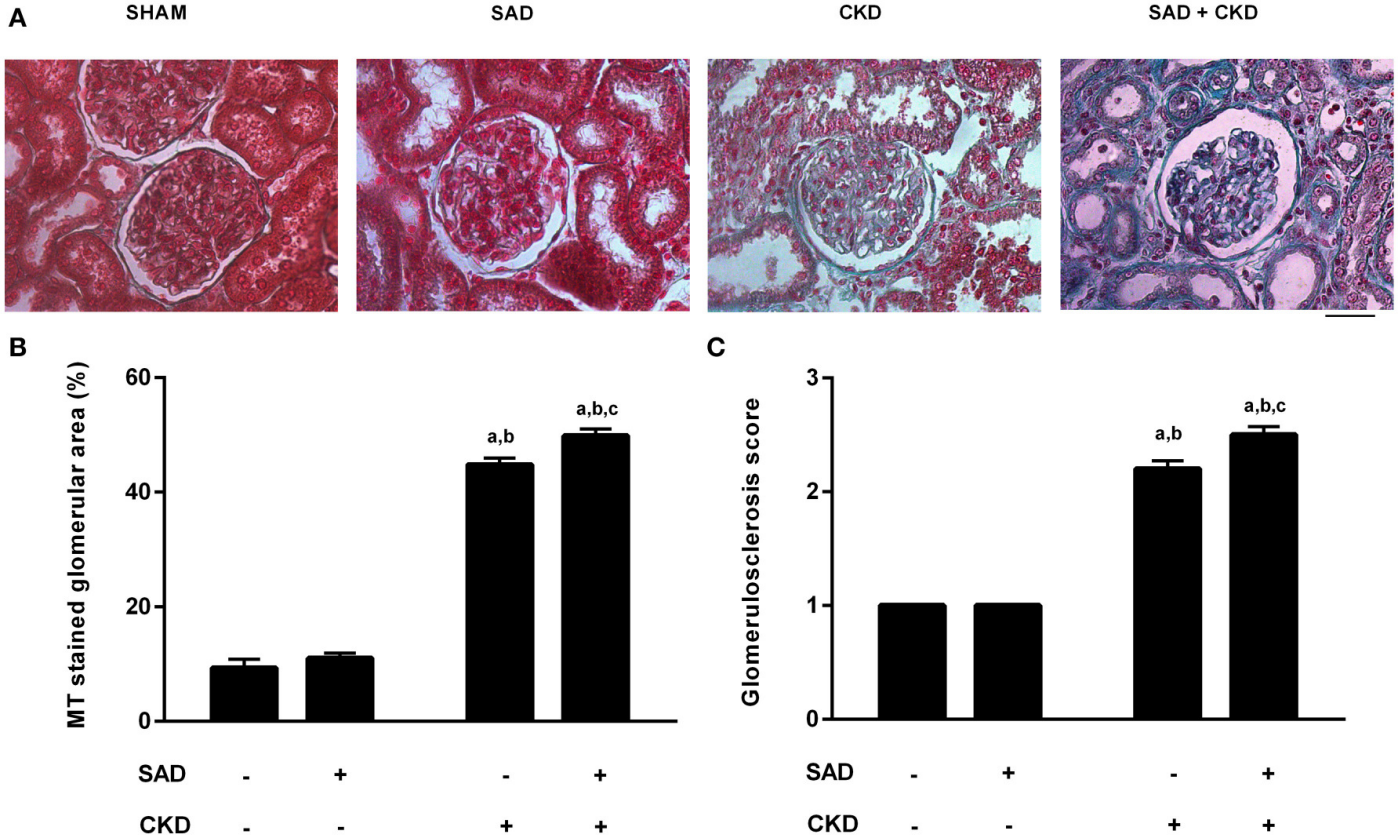
**Glomerular collagen deposition determined using Masson's Trichrome (MT) staining. (A)** Photomicrographs showing collagen deposition (blue) in the glomerulus. Note that glomerulosclerosis is remarkably increased in the SAD+CKD group. **(B)** Percentage area of the glomerulus stained with MT. CKD led to increased collagen deposition, which was more pronounced in the SAD+CKD group **(C)** Glomerulosclerosis score. As expected, CKD animals presented a greater glomerulosclerosis index compared to the sham and SAD groups. The association of SAD+CKD worsened this parameter. The scale bar represents 25 μm. Sham and SAD *n* = 4, CKD and SAD+CKD *n* = 5. Values are means ± SEMs. ^a^*p* < 0.05 vs. Sham group; ^b^*p* < 0.05 vs. SAD group; ^c^*p* < 0.05 vs. CKD group. Two-way ANOVA.

Figure 7 demonstrates the collagen deposition in the glomerulus determined by Masson's Trichrome staining. Typical photomicrographs of all studied groups are displayed in Figure 7A, showing that, as expected, CKD resulted in glomerulosclerosis; however, collagen deposition was further increased when SAD and CKD occurred together. The quantification of the percentage glomerular area stained with Masson's Trichrome (Figure 7B) demonstrates that SAD did not alter glomerular collagen deposition (sham: 9.3 ± 1.5, SAD: 11.0 ± 0.8%). On the other hand, CKD resulted in glomerulosclerosis (44.8 ± 1.2%, *p* < 0.01), which was exacerbated in the SAD+CKD group (49.9 ± 1.2%, *p* < 0.01). The glomerulosclerosis score also confirmed these results. No differences were observed between the sham (1 ± 0) and SAD (1 ± 0) groups. CKD increased the glomerulosclerosis index (2.2 ± 0.07, *p* < 0.01) and the occurrence of SAD and CKD together worsened this parameter (2.5 ± 0.07, *p* < 0.01).

## Discussion

In the past few years, several studies have demonstrated that a variety of diseases, such as atherosclerosis, diabetes and CKD, share a common factor: increased BPV (Roman et al., 2001; Di Iorio et al., 2012; Ushigome et al., 2014). In this scenario, BPV quantification emerges as a possible new marker of target-organ damage. Data from clinical studies demonstrated a significant association between BPV and cardiovascular mortality risk and renal death (Di Iorio et al., 2015). BPV is also correlated with a worse prognosis in cardiovascular and renal diseases (Parati et al., 2012; Di Iorio et al., 2013). However, both experimental and clinical studies have focused in evaluating BPV after the onset of the disease, and it is likely the observed dysfunction may have occurred due to other factors besides increased BPV. In the present study we developed an experimental design that was able to induce increased BPV prior to the onset of kidney disease and our results show that SAD prior to CKD induction worsens renal function, with diminished RBF, increased RVR, as well as augmented uraemia and glomerulosclerosis. Taken together, our results showed that increased BPV may accelerate the progression of CKD, indicating that BPV seems to be an important risk factor to the progression of kidney injury and it should be taken in consideration in the clinical practice.

Although SAD was initially considered a neurogenic hypertension model (Krieger, 1964), it can be considered an excellent experimental model to study the effects of augmented BPV, since the removal of the baroreflex afferent pathway, leads to a diminished BSI and increased BPV with no long-term changes in blood pressure (Su and Miao, 2002). The induction of CKD through 5/6 nephrectomy also resulted in a reduction of BSI and augmented BPV. Similar to our data, Griffin et al. (2004) demonstrated an increased BPV in nephrectomised rats. Clinical studies also showed an augmented BPV and diminished BSI in patients with chronic renal failure (Tozawa et al., 1999; Studinger et al., 2006; Di Iorio et al., 2012). Although CKD animals presented higher values of blood pressure but lower BSI than SAD group, we cannot rule out the effects on hypertension *per se* on BSI. It is well-established in the literature that increased values of blood pressure decrease baroreflex sensitivity in different experimental hypertension models (Vasquez et al., 1994; Peotta et al., 2007; Klippel et al., 2016) and when comparing sham to CKD animals as well as SAD to SAD+CKD group, it appears that increased blood pressure may have contributed to decrease BSI. Another factor that should be considered is the effects of cardiac hypertrophy on BSI. Studies have demonstrated that increased cardiac mass, without hypertension, leads to an impairment of baroreflex function in different animal models (Meyrelles et al., 1998; Gava et al., 2004). In agreement with this, in experimental models of hypertension, the development of baroreflex dysfunction coincides with the onset of cardiac hypertrophy (Head, 1994). In our study, both SAD and CKD groups present cardiac hypertrophy and reduced BSI, and these alterations were further increased in SAD+CKD animals, indicating that, besides the aforementioned factors, cardiac hypertrophy may have contributed to decreased baroreflex sensitivity.

One of the most important findings of our study concerns the association of SAD and CKD. In almost all analyzed parameters, when SAD and CKD were concomitant, the animals displayed worsened function. Occurring together, SAD and CKD resulted in a two-fold higher reduction in BSI and a 1.5-fold higher increase in BPV. The mechanisms involved in these alterations are not fully understood; however, studies have noted changes in sympathetic activity. According to Irigoyen et al. (1995), chronic SAD rats present several short periods of sympathetic hyperactivity, which could contribute to blood pressure fluctuations. Spectral analysis of the arterial pressure demonstrated a rise in the low frequency component in both mice (Fazan et al., 2005) and rats (Mostarda et al., 2010) subjected to SAD, indicating an increase in the sympathetic activity. Comparable to these data, Shan et al. (2004) showed that rats with SAD presented higher levels of noradrenaline in areas involved in the vasomotor control, such as medulla oblongata and hypothalamus. The increase in sympathetic activity may also be involved in augmented BPV in the CKD animals because 5/6 nephrectomy also results in an elevation of circulating catecholamines (Amann et al., 2000; Leineweber et al., 2002). The kidneys present both afferent and efferent sympathetic fibers, and in addition to being the target of sympathetic activity, they may also play a role as the source sympathetic activity (Campese, 2000), including during chronic renal failure. Corroborating this idea, Bigazzi et al. (1994) demonstrated that 5/6 nephrectomised rats present augmented turnover of noradrenaline in the posterior hypothalamic nucleus, which was attenuated after bilateral rhizotomy. Therefore, because sympathetic nervous system hyperactivity is a common feature of both SAD and CKD, the combination of both interventions may lead to even higher sympathetic activation, explaining the reduction in BSI and elevation in BPV found to be more prominent in the SAD+CKD group.

Regarding the haemodynamic parameters, the SAD animals did not demonstrate any changes in arterial pressure nor heart rate. This result is in accordance with the literature because the SAD animals show hypertension only in the initial phase of SAD. In the chronic phase, arterial pressure returns to normal levels, although BPV remains high (Norman et al., 1981). Nephrectomised animals also presented hypertension, which was also an expected result because studies have demonstrated that the infarction of the renal poles results in an elevation of arterial pressure (Griffin et al., 2004). However, the resting values of systolic, diastolic, and MAP, as well as heart rate, were more elevated in the SAD+CKD group. The hyperactivation of the sympathetic nervous system by the combination of SAD and CKD may play a role in the development of these higher levels of hypertension, although we cannot rule out the role of the renin angiotensin system (RAS). Nephrectomised animals present an elevation of plasma (2 weeks after nephrectomy) and tecidual angiotensin II (Mackie et al., 2001; Vaziri et al., 2007). Nishimura et al. (2007) demonstrated that renin, angiotensin-converting enzyme (ACE) and AT1 receptor mRNA expression is elevated in the hypothalamus and brainstem of nephrectomised animals. It is interesting to note that these regions are involved in sympathetic activation, and the increase of tecidual RAS in these areas may play a role in the sympathetic hyperactivity observed in CKD. SAD also leads to an increase in the RAS system. Angiotensin II levels, AT1 receptors mRNA expression and ACE activity are elevated in the heart, aorta and kidney of SAD animals (Miao et al., 2003; Shan et al., 2004; Feng et al., 2011). Because both SAD and CKD result in augmented RAS, once again, the combination of both conditions may be additive and lead to higher levels of hypertension in the SAD+CKD group. In addition, activation of tecidual RAS may also be involved in the cardiac hypertrophy presented in the SAD and the CKD animals. The increase in cardiac weight/body weight ratio was even higher in the SAD+CKD group, and the mechanisms involved in this alteration may include activation of SNS, augmented tecidual RAS and the higher level of hypertension presented by the animals.

In the renal function studies, we observed that SAD *per se* did not produce any changes in the GFR. As expected, CKD animals presented a remarkable reduction in the GFR, and the combination of SAD and CKD did not worse this parameter. However, in the SAD+CKD group, the reduction of renal plasma (RPF) and blood (RBF) flow and the increase in RVR were statistically greater than in all other groups.

The maintenance of the GFR in the SAD+CKD group compared to CKD animals, even with a smaller RBF, may involve the RAS system. It is well-established that angiotensin II is able to cause a preferential constriction of the efferent arteriole, increasing glomerular hydrostatic pressure and maintaining the GFR (Bidani et al., 2013). As both SAD and CKD increase intrarenal angiotensin II (Shan et al., 2004; Vaziri et al., 2007), we can speculate that a greater activation of RAS in SAD+CKD may prevent a greater reduction of GFR in this group. Although SAD+CKD animals did not present a further reduction in GFR, it seems that the combination of both procedures resulted in a worsened renal function, because other analyzed parameters such as hyperuraemia and glomerulosclerosis were exacerbated in this group. Additionally, the correlation analysis showed that there is a negative relationship between GFR and BPV.

Another pathway that might be involved in the observed changes in renal function is the increased sympathetic nerve activity. Even thought we did not quantify renal sympathetic nerve activity in the present investigation, studies have demonstrated that both SAD and CKD result in increased sympathetic drive (Irigoyen et al., 1995; Amann et al., 2000; Leineweber et al., 2002; Fazan et al., 2005). It is well-stablished that SNS activity plays an important role in the genesis of hypertension; however, the deleterious effects of increased sympathetic drive on the kidneys are not only caused by higher blood pressure levels (Joles and Koomans, 2004). Prolonged SNS hyperactivity can damage intrarenal blood vessels by inducing proliferation of smooth muscle cells and fibroblasts in the vessel wall (Zhang and Faber, 2001; Erami et al., 2002). In addition, activation of renal sympathetic fibers result in vasoconstriction and reduce RBF (Johns et al., 2011), leading to renal injury. In our study, it is possible that the combination of SAD and CKD resulted in an even higher sympathetic drive than when these situations occurred alone, contributing to a worsened renal function on SAD+CKD group.

Although we speculate that increased BPV in SAD+CKD may be responsible for the observed changes in renal function, we cannot rule out the role of hypertension, since this group presented higher levels of systolic, diastolic and MAP. However, the effects of elevated BPV in renal organ damage may be more important than the classic risk factors of a high blood pressure. Corroborating this idea, Miao et al. (2006) identified that increased short-term BPV is a more critical determinant for renal damage than mean BP levels. An important clinical study developed by Parati et al. (1987) demonstrated that, for nearly any level of blood pressure, the patients who presented higher BPV also had increased prevalence and severity of organ damage, in both short and long term evaluation of BPV. Cross-sectional studies in non-treated hypertensive patients have found an increased short term BPV to be positively correlated with impaired renal function (Parati et al., 2012). Taken together, these data indicate that, regardless the blood pressure levels, there is a relationship between BPV and the severity of organ damage, including in hypertensive states.

The alterations in renal haemodynamic observed in our study may occur also due to changes in the nitric oxide (NO) system, which plays an important role in regulating RBF (Mattson and Meister, 2005; Toda and Okamura, 2011), especially counterbalancing the effects of increased sympathetic activity. When SNS activity is augmented, NO formation also increases to prevent kidney ischaemia, mainly in medullar levels (Zou and Cowley, 2000). However, a higher NO generation does not necessarily leads to more effects of this molecule, especially if oxidative stress is increased, as observed in the SAD+CKD group. Similar to these data, studies have demonstrated that both experimental SAD and CKD induce a smaller NO bioavailability (Nakayama et al., 2009; Wu et al., 2013). Our data shows that SAD+CKD animals present higher oxidative stress in the blood, indicating that renal oxidative could also be increased. Therefore, the greater increase in RVR and, consequently, the reduction in RBF, may be attributed to the augmented oxidative stress observed in the SAD+CKD group, reducing NO bioavailability. Thus, the prominent effects of the association of SAD and CKD on RBF and RVR may be attributed to activation of renal vasoconstrictive systems (SNS and SRA) and reduced NO bioavailability.

The reduction of NO bioavailability may also have played a role in the enhanced levels of glomerulosclerosis presented by the SAD+CKD rats. Corroborating this hypothesis, previous studies have demonstrated that eNOS ^−/−^ mice exhibit exacerbated renal interstitial injury and global glomerulosclerosis (Nakayama et al., 2009) and that iNOS ^−/−^ mice show higher levels of tubular apoptosis (Miyajima et al., 2001) and interstitial fibrosis (Hochberg et al., 2000). In the kidney, NO reduced mesangial proliferation and extracellular matrix synthesis (Zhou et al., 2004) and the mechanisms involved in these effects include inhibition of TGF-β_1_ and its downstream effector molecule fibronectin (Zhou et al., 2008), inhibition of TNF-α (Whiting et al., 2013) and modulation of cytokine-induced metalloproteinases and inhibitors of metalloproteinases (Yang et al., 2008).

Taken together, these data indicate that increased BPV prior to renal dysfunction induced by SAD, as well as hypertension, exacerbates renal injury, and glomerulosclerosis in an experimental model of CKD. These results reinforce the role of increased BPV and hypertension as important markers to the progression of renal diseases.

## Author contributions

FFF performed experiments and drafted manuscript, GA performed experiments, MP performed experiments, FPF performed experiments, JG performed experiments, CB analyzed data, interpreted results of experiments, edited and revised manuscript, EC analyzed data, interpreted results of experiments, edited and revised manuscript, SM analyzed data, interpreted results of experiments, edited and revised manuscript, AG conception and design of research, analyzed data, interpreted results of experiments, prepared figures, drafted manuscript, edited and revised manuscript, approved final version of manuscript.

### Conflict of interest statement

The authors declare that the research was conducted in the absence of any commercial or financial relationships that could be construed as a potential conflict of interest.
